# Diet and stones: Associations from a large, population-representative study of urolithiasis and renal colic-like pain symptoms in Poland

**DOI:** 10.1371/journal.pone.0333733

**Published:** 2026-02-03

**Authors:** Jakub Szymanski, Przemyslaw Dudek, Pawel Rajwa, Wojciech Krajewski, Piotr Bryniarski, Katarzyna Krzanowska, Piotr Chlosta, Mikolaj Przydacz

**Affiliations:** 1 Department of Urology, Jagiellonian University Medical College, Krakow, Poland; 2 Second Department of Urology, Centre of Postgraduate Medical Education, Warsaw, Poland; 3 Department of Urology and Oncologic Urology, Wroclaw Medical University, Wroclaw, Poland; 4 Department of Urology, Division of Medical Sciences in Zabrze, Medical University of Silesia in Katowice, Zabrze, Poland; 5 Department of Nephrology, Jagiellonian University Medical College, Krakow, Poland; Metrohealth Medical Center, UNITED STATES OF AMERICA

## Abstract

**Introduction:**

Urolithiasis and renal colic-like pain symptoms are prevalent conditions influenced by many factors, including diet. Regional assessments, particularly in culturally distinct areas, are essential for understanding specific risk patterns and for developing targeted public health strategies and educational programs. Therefore, the aim of this study was to identify associations between urolithiasis, renal colic-like pain symptoms, and diet, including body weight, in a representative sample of adult Poles.

**Methods:**

A population-representative online survey of 10,029 adults was conducted using census-based quota sampling to ensure balanced representation by age, sex, and residence across all 16 Polish voivodships, including urban and rural areas.

**Results:**

Normal-weight individuals had the lowest prevalence of urolithiasis and renal colic-like pain symptoms; overweight, obese, and underweight persons had higher prevalences (p < 0.001). Eighteen food items were linked to urolithiasis, with beef, legumes, soy, soda, coffee, and fast food found as independent predictors of urolithiasis. Nineteen foods were associated with renal colic-like pain symptoms, with processed meat, soy, legumes, fruit juices, soda, instant meals, and fast food identified as independent predictors of symptoms. Fast food showed the strongest effects, with daily consumption nearly tripling the risk of urolithiasis (OR=2.847; p = 0.001) and increasing renal colic-like pain symptoms by 64.5% (OR=1.645; p = 0.006) compared with no consumption of fast food.

**Conclusions:**

This study provides the first comprehensive, population-based analysis of urolithiasis, renal colic-like pain symptoms, and dietary patterns in Central and Eastern Europe. Our findings demonstrate that both conditions are influenced by body weight and a range of dietary factors.

## Introduction

Stone disease, or urolithiasis, is characterized by the formation of calculi within the urinary tract. Stone disease is a major global health issue, although prevalence varies from 1% to 20% across populations because of complex interactions between factors such as geography, climate, genetics, lifestyle, and importantly diet [1]. Recent studies indicate a worldwide increase in urolithiasis prevalence, largely driven by dietary changes and the rising incidence of metabolic conditions such as obesity [2–4]. Therefore, dietary factors have a fundamental influence on formation of urinary stones. Diet affects the composition of the urine and may contribute to the supersaturation of the stone-forming salt, thereby influencing the risk of stone formation [5,6].

Urolithiasis can result in a range of consequences, from mild discomfort such as renal colic, to long-term complications such as hypertension, hydronephrosis and chronic kidney disease [5–7]. Additionally, urolithiasis may contribute to psychological distress, including anxiety and depression, and to economic and lifestyle challenges, such as increased medical expenses and reduced work productivity [8,9]. Consequently, urolithiasis places a significant financial burden on national healthcare systems. For example, in the United Kingdom, the cost of treating this condition is comparable to the combined expenditure for bladder and prostate cancer [10]. In the United States, the annual healthcare costs associated with urolithiasis management are projected to reach $30 billion by 2030 [11]. Therefore, a thorough understanding of the key factors contributing to urolithiasis is essential for developing effective and targeted prevention strategies and health-related interventions. This understanding is particularly important for modifiable risk factors, such as dietary habits, for which modifications could be essential in reducing the prevalence of urolithiasis.

Despite the multifaceted burden of urolithiasis, there is a scarcity of large and reliable population-level studies of the relation between dietary habits and the prevalence of urolithiasis and renal colic-like pain symptoms. Indeed, experts emphasize that high-quality research should rely on surveys conducted within representative populations to ensure robust findings [12]. Moreover, there is a particularly pronounced lack of such data for Central and Eastern Europe. The distinct cultural, regional, and ethnic characteristics of the Slavic population pose challenges in directly comparing findings with those from Western European or American populations [13]. Therefore, regional assessments are essential for drawing accurate conclusions, deepening the understanding of urolithiasis, and identifying key contributing factors, particularly dietary patterns that may differ from those of other populations. Given these considerations, we aimed to provide a comprehensive analysis of the associations between urolithiasis, renal colic-like pain symptoms, and dietary factors, including body weight, in a population-representative sample of adults aged 18 years and older from all regions of Poland.

## Methods

This study builds upon data from the POLSTONE study, a population-based, cross-sectional epidemiological investigation assessing the prevalence of urolithiasis and renal colic symptoms in Poland. The POLSTONE study’s conceptual framework, design, methodology, and data collection are detailed [14,15]; thus, only a brief summary is provided here. The Research Ethics Committee of Jagiellonian University Medical College in Krakow, Poland approved this study (118.6120.94.2023), which is also registered with ClinicalTrials.gov (NCT06176469). Before participation, all individuals provided informed consent and were thoroughly briefed on the study’s duration, the nature of data collection, data storage protocols, investigator details, and research objectives.

### Study design

The POLSTONE study included a representative cohort of men and women aged 18 years and older from both urban and rural areas across all regions of Poland. The study was conducted via computer-assisted web interviews; in 2022, 93.33% of households in Poland had internet access, with minimal differences between urban and rural areas [16]. Given this high internet penetration, conducting an online survey was deemed an effective approach for a population-level study. The survey was administered by a research agency (4P) holding international quality certifications, and responses were collected on a secure web server with Secure Socket Layer encryption. To ensure data quality, regular stratification checks were performed, and post-stratification weights were applied based on age, sex, and place of residence to adjust for response rate imbalances. The survey took place between November 1, 2023, and January 15, 2024. This timeframe was specifically chosen outside the high-temperature season to reduce potential biases in the prevalence of renal colic-like symptoms; a 24%−84% increase in these symptoms is associated with high temperature [17,18]. The study sample was structured based on the most recent population census data (2021) [19], and we employed a proportionate quota sampling method to ensure representativeness in terms of age, sex, and place of residence across all 16 Polish voivodships. Urban and rural classifications followed definitions provided by the Central Statistical Office of Poland [20].

### Measures

General sociodemographic data were collected from all respondents. Participants were asked about a history of urolithiasis with the question: “*Have you ever been diagnosed with urinary tract stones/urolithiasis?*” Then, renal colic-like pain symptoms were assessed with the question: “*Have you ever experienced back pain (in the kidney area) that was cramp-like, intermittent, coming and going, and possibly radiating to the groin (commonly referred to as renal colic)? This pain is typically located along the sides of the back and is unrelated to movement or changes in body position. The pain may also be accompanied by nausea or vomiting.*” Respondents self-reported whether they had been diagnosed with or considered being overweight or obese. They also supplied their weight and height for body mass index (BMI) calculation. Based on BMI, they were categorized into four groups: underweight (<18.49), normal weight (18.5–24.99), overweight (25–29.9), and obese (>30). Additionally, they answered questions about the consumption of 23 specific foods. Dietary habits were assessed based on the reported frequency of consumption, categorized as “every day,” “every other day,” “once a week,” “rarely,” or “never.”

### Statistics

Descriptive statistics for quantitative variables included the mean, standard deviation, median, quartiles, and range. For qualitative variables, absolute (n) and relative (%) frequencies were reported. Group comparisons for qualitative variables were performed using the chi-squared test with Yates correction for 2x2 tables or Fisher’s exact test when expected values were low. The Mann-Whitney test was applied to compare quantitative variables between two groups, whereas the Kruskal-Wallis test followed by the post-hoc Dunn test were used for comparisons involving three or more groups. Multiple logistic regression was used to assess the potential influence of predictors on a dichotomous variable, with results presented as odds ratios and 95% confidence intervals. A significance threshold of 0.05 was used, and all statistical analyses were conducted using R software, version 4.4.1.

## Results

### Participant demographics

The survey included 10,029 respondents, representing the general Polish population. The cohort had more women (n = 5,249) than men (n = 4,780). The majority of participants had a secondary level of education (n = 7,093), were employed (n = 5,409), and were married (n = 5,281). Additionally, urban residents (n = 5,281) outnumbered rural area residents. The overall response rate for the survey was 23.1%.

### Urolithiasis

Respondents who self-reported being overweight or obese had a higher prevalence of urolithiasis compared with persons who did not report these conditions (p < 0.001, Table 1). Considering BMI, the highest prevalence of urolithiasis was observed in individuals with obesity, reaching 15.27%, whereas the lowest prevalence (10.76%) was found for the normal-weight group (p < 0.001, Table 1).

**Table 1:** Association of urolithiasis with body weight.

Parameter	Group	Urolithiasis		p
		No	Yes	
Overweight/Obesity	No (N = 6804)	6026 (88.57%)	778 (11.43%)	p < 0.001*
	Yes (N = 3225)	2714 (84.16%)	511 (15.84%)	
Body Mass Index (BMI)	Underweight (N = 275)	242 (88.00%)	33 (12.00%)	p < 0.001*
	Normal weight (N = 3967)	3540 (89.24%)	427 (10.76%)	
	Overweight (N = 3569)	3076 (86.19%)	493 (13.81%)	
	Obesity (N = 2056)	1742 (84.73%)	314 (15.27%)	

p – chi-square test or Fisher’s exact test.

*statistically significant difference (p < 0.05).

We observed a higher prevalence of urolithiasis in respondents who consumed beef, pork, dark bread, legumes, soy products, nuts, spinach, strawberries, fruit juices, soda, coffee, instant meals (e.g., Chinese soups), and fast food dishes compared with persons who did not report consuming one or more of these products (p < 0.01, S1 Table). Conversely, a lower prevalence of urolithiasis was observed for respondents who consumed poultry, processed meat (cold cuts, sausages, frankfurters, pâtés, canned meats), diary, white bread and tea compared with individuals who did not report consuming one or more of these products (p < 0.05, S1 Table). We did not find associations between prevalence of urolithiasis and consumption of cereal products, fresh fruits, fresh vegetables, cocoa, chocolate, and highly processed sweets.

Multivariate regression analysis confirmed an increased risk of urolithiasis associated with the consumption of beef, legumes, soy products, soda, coffee, and fast food dishes (Table 2). The highest risk was observed for fast food consumption, whereby eating fast food daily nearly tripled the risk of urolithiasis (OR = 2.847) compared with not ever consuming fast food. Conversely, the model indicated a decreased risk of urolithiasis associated with consuming poultry, spinach, strawberries, tea, and instant meals (e.g., Chinese soups) (Table 2). The greatest risk reduction was observed for poultry consumption, as eating poultry once a week reduced the risk of urolithiasis by 59.6% (OR = 0.404) compared with not consuming poultry.

**Table 2:** Multivariate logistic regression analysis of dietary habits and urolithiasis.

Factor		N	n	OR	95%CI		p
Beef	Never	1422	156	1	ref.		
	Rarely	5588	688	1.283	1.023	1.61	0.031*
	Once in a week	2194	267	1.139	0.874	1.485	0.335
	Every other day	489	83	1.212	0.829	1.772	0.321
	Every day	336	95	1.222	0.806	1.853	0.346
Pork	Never	405	62	1	ref.		
	Rarely	2126	246	0.973	0.618	1.533	0.906
	Once in a week	4655	554	0.979	0.621	1.543	0.928
	Every other day	2427	358	1.146	0.715	1.837	0.572
	Every day	416	69	0.828	0.464	1.477	0.524
Poultry	Never	249	48	1	ref.		
	Rarely	885	123	0.477	0.285	0.799	0.005*
	Once in a week	4457	527	0.404	0.247	0.661	<0.001*
	Every other day	3876	496	0.457	0.278	0.751	0.002*
	Every day	562	95	0.558	0.318	0.978	0.042*
Processed meats (cold cuts, sausages, frankfurters, pâtés, canned meats)	Never	333	55	1	ref.		
	Rarely	1184	178	1.12	0.695	1.805	0.642
	Once in a week	2258	303	0.908	0.56	1.472	0.695
	Every other day	3489	437	0.864	0.533	1.401	0.554
	Every day	2765	316	0.782	0.477	1.283	0.331
Dairy	Never	97	22	1	ref.		
	Rarely	418	61	0.584	0.3	1.136	0.113
	Once in a week	1549	216	0.683	0.368	1.268	0.228
	Every other day	3237	414	0.742	0.402	1.371	0.341
	Every day	4728	576	0.728	0.395	1.343	0.31
Grain products	Never	238	37	1	ref.		
	Rarely	1433	193	1.001	0.63	1.592	0.996
	Once in a week	2515	311	0.751	0.472	1.194	0.226
	Every other day	2469	320	0.804	0.504	1.284	0.361
	Every day	3374	428	0.79	0.494	1.262	0.324
White bread	Never	560	79	1	ref.		
	Rarely	1419	194	1.01	0.727	1.403	0.952
	Once in a week	1270	204	1.152	0.817	1.624	0.42
	Every other day	2116	297	1.157	0.828	1.616	0.393
	Every day	4664	515	0.94	0.678	1.305	0.713
Dark bread	Never	685	82	1	ref.		
	Rarely	2733	284	0.917	0.673	1.25	0.585
	Once in a week	2025	249	0.902	0.65	1.252	0.537
	Every other day	2303	324	0.945	0.68	1.312	0.734
	Every day	2283	350	0.994	0.715	1.382	0.973
Legumes	Never	603	57	1	ref.		
	Rarely	4750	553	1.498	1.061	2.115	0.022*
	Once in a week	3625	459	1.484	1.031	2.136	0.034*
	Every other day	818	153	1.773	1.165	2.698	0.007*
	Every day	233	67	2.119	1.217	3.69	0.008*
Soy products	Never	3703	421	1	ref.		
	Rarely	4480	509	1.008	0.856	1.187	0.924
	Once in a week	1236	214	1.401	1.096	1.791	0.007*
	Every other day	435	87	1.303	0.908	1.869	0.15
	Every day	175	58	1.62	0.961	2.731	0.07
Fresh Fruits	Never	108	18	1	ref.		
	Rarely	902	106	1.218	0.573	2.588	0.609
	Once in a week	1998	253	1.149	0.548	2.408	0.713
	Every other day	2868	345	1.089	0.518	2.287	0.823
	Every day	4153	567	1.074	0.511	2.258	0.851
Fresh vegetables	Never	95	16	1	ref.		
	Rarely	849	107	0.974	0.435	2.182	0.949
	Once in a week	2097	274	0.989	0.447	2.192	0.979
	Every other day	3281	393	0.902	0.407	2.001	0.8
	Every day	3707	499	0.985	0.443	2.19	0.97
Nuts	Never	714	93	1	ref.		
	Rarely	4058	474	0.927	0.695	1.236	0.607
	Once in a week	3048	390	0.798	0.587	1.084	0.149
	Every other day	1321	179	0.741	0.525	1.046	0.089
	Every day	888	153	0.922	0.641	1.328	0.663
Cocoa. Chocolate	Never	452	68	1	ref.		
	Rarely	3335	405	0.969	0.686	1.367	0.856
	Once in a week	3657	475	1.008	0.708	1.434	0.967
	Every other day	1788	227	0.834	0.571	1.217	0.347
	Every day	797	114	0.821	0.534	1.261	0.367
Highly-processed sweets	Never	567	84	1	ref.		
	Rarely	3056	363	0.955	0.695	1.313	0.778
	Once in a week	3461	458	1.02	0.734	1.418	0.906
	Every other day	2094	267	1.054	0.743	1.497	0.768
	Every day	851	117	1.024	0.679	1.545	0.909
Spinach	Never	2282	293	1	ref.		
	Rarely	5038	590	0.866	0.726	1.034	0.112
	Once in a week	2099	276	0.762	0.606	0.957	0.019*
	Every other day	466	87	0.722	0.496	1.049	0.087
	Every day	144	43	0.657	0.348	1.237	0.193
Strawberries	Never	463	66	1	ref.		
	Rarely	7002	810	0.786	0.567	1.09	0.149
	Once in a week	1744	260	0.776	0.542	1.112	0.167
	Every other day	564	100	0.743	0.485	1.138	0.172
	Every day	256	53	0.52	0.293	0.922	0.025*
Fruit juices	Never	686	90	1	ref.		
	Rarely	3309	375	1.005	0.751	1.344	0.976
	Once in a week	3043	390	1.081	0.798	1.464	0.617
	Every other day	1939	272	1.092	0.794	1.502	0.587
	Every day	1052	162	1.216	0.858	1.722	0.272
Sweet beverages	Never	2339	307	1	ref.		
	Rarely	3737	427	1.144	0.943	1.387	0.172
	Once in a week	2088	279	1.244	0.983	1.575	0.069
	Every other day	1084	161	1.431	1.078	1.898	0.013*
	Every day	781	115	1.517	1.097	2.096	0.012*
Coffee	Never	972	95	1	ref.		
	Rarely	661	77	1.268	0.882	1.822	0.2
	Once in a week	635	129	1.995	1.397	2.847	<0.001*
	Every other day	787	130	1.455	1.042	2.031	0.028*
	Every day	6974	858	1.196	0.922	1.553	0.178
Tea	Never	314	56	1	ref.		
	Rarely	876	108	0.782	0.518	1.18	0.241
	Once in a week	902	141	0.774	0.509	1.176	0.23
	Every other day	1268	175	0.764	0.513	1.137	0.184
	Every day	6669	809	0.684	0.477	0.98	0.039*
Instant meals	Never	3730	523	1	ref.		
	Rarely	4080	441	0.831	0.701	0.985	0.033*
	Once in a week	1544	190	0.741	0.578	0.951	0.019*
	Every other day	478	81	0.731	0.502	1.064	0.102
	Every day	197	54	0.944	0.548	1.626	0.835
Fast food	Never	2301	330	1	ref.		
	Rarely	5409	604	1.127	0.936	1.358	0.207
	Once in a week	1763	224	1.264	0.968	1.651	0.085
	Every other day	429	83	1.621	1.082	2.429	0.019*
	Every day	127	48	2.847	1.527	5.311	0.001*

p – multivariate logistic regression.

*statistically significant dependence (p < 0.05).

### Renal colic-like pain symptoms

Similar to urolithiasis, respondents who self-reported being overweight or obese had a higher prevalence of renal colic-like pain symptoms compared with persons who did not report these conditions (p < 0.001, Table 3). Considering BMI, the highest prevalence of renal colic-like pain symptoms was observed for obese individuals (48.05%), whereas the lowest prevalence (40.74%) was found for the normal-weight group (p < 0.001, Table 3).

**Table 3:** Association of renal colic-like pain symptoms with body weight.

Parameter	Group	Renal colic-like pain symptoms		p
		No	Yes	
Overweight/Obesity	No (N = 6804)	4028 (59.20%)	2776 (40.80%)	p < 0.001*
	Yes (N = 3225)	1684 (52.22%)	1541 (47.78%)	
Body Mass Index (BMI)	Underweight (N = 275)	145 (52.73%)	130 (47.27%)	p < 0.001*
	Normal weight (N = 3967)	2351 (59.26%)	1616 (40.74%)	
	Overweight (N = 3569)	2064 (57.83%)	1505 (42.17%)	
	Obesity (N = 2056)	1068 (51.95%)	988 (48.05%)	

p – chi-square test or Fisher’s exact test.

*statistically significant difference (p < 0.05).

We observed a higher prevalence of renal colic-like pain symptoms in respondents who consumed beef, pork, poultry, processed meat (cold cuts, sausages, frankfurters, pâtés, canned meats), white bread, dark bread, legumes, soy products, highly processed sweets, spinach, strawberries, fruit juices, soda, coffee, instant meals (e.g., Chinese soups), and fast food dishes, compared with individuals who did not report consuming one or more of these products (p < 0.01, S2 Table). Conversely, a lower prevalence of renal colic-like pain symptoms was observed for respondents who consumed cereal products, fresh fruits, and tea compared to those who did not report consuming one or more of these products (p < 0.05, S2 Table). We did not find associations between prevalence of renal colic-like pain symptoms and consuming diary, fresh vegetables, nuts, cocoa, and chocolate.

Multivariate regression analysis confirmed an increased risk of renal colic-like pain symptoms associated with the consumption of processed meat (cold cuts, sausages, frankfurters, pâtés, canned meats), legumes, soy products, fruit juices, soda, instant meals (e.g., Chinese soups), and fast food dishes (Table 4). The highest risk was observed for fast food consumption, whereby eating fast food daily increased the risk of renal colic-like pain symptoms by 64.5% (OR = 1.645) compared with not consuming fast food at all. Interestingly, our model did not identify any foods associated with a decreased risk of renal colic-like pain symptoms.

**Table 4:** Multivariate logistic regression analysis of dietary habits and renal colic-like pain symptoms.

Factor		N	n	OR	95%CI		p
Beef	Never	1422	565	1	ref.		
	Rarely	5588	2293	1.059	0.919	1.221	0.428
	Once in a week	2194	989	1.063	0.899	1.256	0.478
	Every other day	489	272	1.282	0.993	1.655	0.057
	Every day	336	198	0.879	0.646	1.198	0.415
Pork	Never	405	153	1	ref.		
	Rarely	2126	843	0.938	0.688	1.279	0.686
	Once in a week	4655	1990	0.968	0.71	1.32	0.838
	Every other day	2427	1125	0.991	0.718	1.369	0.958
	Every day	416	206	0.856	0.58	1.264	0.434
Poultry	Never	249	91	1	ref.		
	Rarely	885	365	0.812	0.548	1.203	0.299
	Once in a week	4457	1825	0.748	0.512	1.093	0.133
	Every other day	3876	1724	0.79	0.539	1.157	0.226
	Every day	562	312	1.012	0.664	1.541	0.957
Processed meats (cold cuts, sausages, frankfurters, pâtés, canned meats)	Never	333	111	1	ref.		
	Rarely	1184	484	1.501	1.054	2.138	0.024*
	Once in a week	2258	965	1.424	1	2.027	0.05*
	Every other day	3489	1495	1.484	1.043	2.111	0.028*
	Every day	2765	1262	1.518	1.06	2.173	0.023*
Dairy	Never	97	45	1	ref.		
	Rarely	418	187	0.903	0.534	1.528	0.704
	Once in a week	1549	703	0.903	0.549	1.485	0.687
	Every other day	3237	1356	0.858	0.523	1.407	0.545
	Every day	4728	2026	0.922	0.563	1.51	0.748
Grain products	Never	238	105	1	ref.		
	Rarely	1433	619	0.933	0.678	1.283	0.67
	Once in a week	2515	1127	0.905	0.659	1.244	0.54
	Every other day	2469	1085	0.888	0.644	1.225	0.47
	Every day	3374	1381	0.799	0.58	1.102	0.172
White bread	Never	560	213	1	ref.		
	Rarely	1419	538	0.849	0.674	1.068	0.162
	Once in a week	1270	595	0.952	0.748	1.212	0.692
	Every other day	2116	903	0.901	0.714	1.138	0.382
	Every day	4664	2068	0.929	0.74	1.166	0.525
Dark bread	Never	685	301	1	ref.		
	Rarely	2733	1101	0.854	0.703	1.038	0.113
	Once in a week	2025	956	1.074	0.873	1.322	0.5
	Every other day	2303	1002	0.972	0.787	1.201	0.795
	Every day	2283	957	0.969	0.783	1.201	0.776
Legumes	Never	603	260	1	ref.		
	Rarely	4750	1967	0.982	0.801	1.203	0.859
	Once in a week	3625	1530	0.956	0.769	1.189	0.685
	Every other day	818	411	1.021	0.78	1.336	0.88
	Every day	233	149	1.617	1.075	2.433	0.021*
Soy products	Never	3703	1481	1	ref.		
	Rarely	4480	1873	1.047	0.942	1.164	0.394
	Once in a week	1236	616	1.321	1.117	1.561	0.001*
	Every other day	435	232	1.083	0.829	1.413	0.559
	Every day	175	115	1.302	0.842	2.013	0.235
Fresh Fruits	Never	108	51	1	ref.		
	Rarely	902	396	1.069	0.622	1.837	0.81
	Once in a week	1998	915	1.081	0.632	1.848	0.776
	Every other day	2868	1233	1.072	0.626	1.837	0.799
	Every day	4153	1722	1.141	0.665	1.957	0.633
Fresh vegetables	Never	95	42	1	ref.		
	Rarely	849	381	1.039	0.59	1.83	0.894
	Once in a week	2097	942	1.046	0.597	1.832	0.876
	Every other day	3281	1404	1.011	0.577	1.774	0.969
	Every day	3707	1548	1.009	0.574	1.774	0.976
Nuts	Never	714	310	1	ref.		
	Rarely	4058	1768	1.025	0.849	1.238	0.794
	Once in a week	3048	1312	0.904	0.739	1.106	0.328
	Every other day	1321	553	0.848	0.675	1.065	0.156
	Every day	888	374	0.924	0.72	1.186	0.537
Cocoa. Chocolate	Never	452	197	1	ref.		
	Rarely	3335	1383	1.01	0.797	1.28	0.933
	Once in a week	3657	1578	1.046	0.821	1.333	0.715
	Every other day	1788	790	1.034	0.799	1.338	0.798
	Every day	797	369	1.096	0.821	1.465	0.534
Highly-processed sweets	Never	567	228	1	ref.		
	Rarely	3056	1208	0.881	0.704	1.102	0.267
	Once in a week	3461	1494	0.863	0.685	1.088	0.212
	Every other day	2094	955	0.872	0.683	1.112	0.269
	Every day	851	432	0.955	0.722	1.262	0.745
Spinach	Never	2282	978	1	ref.		
	Rarely	5038	2076	0.952	0.846	1.071	0.412
	Once in a week	2099	916	0.923	0.794	1.073	0.296
	Every other day	466	256	1.046	0.805	1.357	0.738
	Every day	144	91	0.698	0.427	1.142	0.152
Strawberries	Never	463	204	1	ref.		
	Rarely	7002	2927	0.886	0.707	1.11	0.292
	Once in a week	1744	769	0.808	0.63	1.036	0.092
	Every other day	564	285	0.844	0.625	1.139	0.267
	Every day	256	132	0.68	0.46	1.006	0.054
Fruit juices	Never	686	247	1	ref.		
	Rarely	3309	1335	1.15	0.943	1.402	0.168
	Once in a week	3043	1317	1.203	0.978	1.48	0.081
	Every other day	1939	913	1.232	0.991	1.533	0.061
	Every day	1052	505	1.296	1.021	1.645	0.033*
Sweet beverages	Never	2339	847	1	ref.		
	Rarely	3737	1524	1.055	0.926	1.202	0.42
	Once in a week	2088	963	1.079	0.922	1.263	0.343
	Every other day	1084	553	1.232	1.019	1.49	0.031*
	Every day	781	430	1.342	1.086	1.66	0.007*
Coffee	Never	972	377	1	ref.		
	Rarely	661	281	1.017	0.813	1.273	0.882
	Once in a week	635	327	1.247	0.983	1.582	0.069
	Every other day	787	383	1.133	0.912	1.408	0.259
	Every day	6974	2949	1.094	0.934	1.28	0.266
Tea	Never	314	134	1	ref.		
	Rarely	876	388	1.13	0.84	1.52	0.42
	Once in a week	902	430	1.074	0.793	1.455	0.644
	Every other day	1268	599	1.161	0.869	1.551	0.312
	Every day	6669	2766	1.04	0.796	1.358	0.773
Instant meals	Never	3730	1359	1	ref.		
	Rarely	4080	1770	1.102	0.984	1.234	0.091
	Once in a week	1544	768	1.101	0.94	1.29	0.231
	Every other day	478	294	1.439	1.116	1.857	0.005*
	Every day	197	126	1.263	0.849	1.878	0.249
Fast food	Never	2301	803	1	ref.		
	Rarely	5409	2292	1.215	1.067	1.383	0.003*
	Once in a week	1763	871	1.252	1.049	1.494	0.013*
	Every other day	429	261	1.339	1.004	1.787	0.047*
	Every day	127	90	1.645	1.42	2.997	0.006*

p – multivariate logistic regression.

*statistically significant dependence (p < 0.05).

## Discussion

This study is the first analysis from Central and Eastern Europe to specifically identify associations between the prevalence of urolithiasis, renal colic-like pain symptoms, and diet, including body weight. Notably, the analysis included a population-representative sample of men and women aged 18 and older from all geographical regions of Poland, ensuring appropriate representation of both urban and rural areas. Our findings indicate that urolithiasis and renal colic-like pain symptoms correlated with body weight, as defined by BMI, and multiple dietary factors. These data are crucial because specific dietary adjustments can significantly contribute to reducing urolithiasis prevalence [21], beyond the general recommendation of a well-balanced diet that includes all food groups as the most beneficial approach [22–24].

Our results indicate that being overweight or obese was associated with the highest prevalence of urolithiasis and renal colic symptoms. Siener et al. explained that overweight and obesity significantly increase the risk of stone formation because of higher urinary excretion of promoters, but not inhibitors, of calcium oxalate stone formation [25]. In a recent systematic review and meta-analysis, Emami et al. examined the link between obesity and the risk of kidney stone formation [26]. The analysis included 15 observational studies published between 2005 and 2022. Results showed that people with obesity had a 1.35 times greater risk of developing kidney stones compared with persons without obesity (95% CI: 1.20–1.52, p < 0.001). Regionally, the association was particularly pronounced in North America and Europe, where the risk was significantly higher than in Asia. Additionally, in a cross-sectional survey-based study in China, Chen et al. demonstrated that individuals with metabolically healthy obesity still had an elevated risk of kidney stones; thus, obesity alone, even in the absence of metabolic abnormalities, can contribute to stone formation [27].

Conversely, we also found that underweight individuals had a higher prevalence of urolithiasis and renal colic-like pain symptoms compared with persons having normal weight. Low body weight may predispose individuals to nutritional deficiencies and electrolyte imbalances, such as hypokalemia [28]. These abnormalities cause metabolic disturbances such as reduced urine output and increased urinary ammonium excretion due to hypophosphaturia and diarrhea with hyperchloremic acidosis which promote kidney stone formation, mainly of ammonium urate. Additionally, malnourished individuals, particularly those with anorexia nervosa, are at risk of developing refeeding syndrome, a condition characterized by severe electrolyte shifts during nutritional rehabilitation [29]. In a population-based retrospective cohort study, Denburg et al. found that young women with anorexia nervosa had more than a threefold higher risk of developing urolithiasis compared with their unexposed counterparts [30]. Although the association between low body weight and urolithiasis is well-documented in the context of eating disorders such as anorexia nervosa, there are only limited studies specifically focused on underweight individuals who do not have eating disorders. Therefore, our study is the first population-based analysis to reliably document the relation between underweight status and the occurrence of urolithiasis or renal colic-like pain symptoms. Finally, our findings further support the conclusion that maintaining a healthy body weight is an important factor in preventing both urolithiasis and renal colic-like pain symptoms.

The intake of animal protein contributes to a high purine load, which leads to increased uric acid production and excretion, factors associated with a higher risk of kidney stone formation [31,32]. High protein intake is also linked to an increased acid load, which elevates urinary calcium levels while reducing urine pH and citrate excretion [33]. Restricting animal protein intake is one of the general preventive measures for urolithiasis outlined in the European Association of Urology Guidelines on Urolithiasis [21]. Our study confirmed that high meat consumption, particularly beef, was strongly correlated with the prevalence of both urolithiasis and renal colic-like pain symptoms. However, an interesting finding from our multivariate analysis showed that poultry consumption appeared to have a protective effect on urolithiasis, although this effect was not observed for renal colic-like pain symptoms. The observed differences in the effects of various meats on urolithiasis may be attributed to several factors, including their purine content, which influences uric acid metabolism, and their impact on urinary pH and citrate excretion. Poultry has a different amino acid profile compared with beef and other meat types, which could influence urinary stone risk factors [34]. However, there are limited studies of the relation between the amino acid composition of different meats and their direct effects on urolithiasis. The specific effects of different meat types on urinary pH and citrate levels may also differ, potentially influencing their association with urolithiasis [35]. In a study by Best et al., persons who consumed poultry had higher urine pH and lower urinary oxalate levels compared with persons who consumed beef [35]. Consequently, the mechanisms of the protective effect of poultry consumption on urolithiasis that we observed is only a hypothesis. Nevertheless, our study underscores the fact that different types of meat have different effects on urolithiasis. Therefore, additional research should be focused on the specific impact of different types of meat on urolithiasis, instead of treating meat consumption as a single category. As experts have suggested, the source of protein matters because various protein types may influence urolithiasis risk differently [31].

In our study, the consumption of grain products (e.g., rice, pasta, cereals, groats, oatmeal) was inversely correlated with renal colic-like pain symptoms and not significantly correlated with urolithiasis. However, the prevalence of urolithiasis was higher among persons who consumed dark bread, and the prevalence of renal colic-like pain symptoms was higher in individuals who consumed both dark and white bread. This finding aligns with the results of Shabani et al., who identified bread consumption as an independent risk factor for urolithiasis [36]. To explain why bread has effects on urolithiasis and renal colic-like pain symptoms different from other grain products, we can consider several potential factors that focus on the unique characteristics of bread consumption compared with other grain-based foods such as rice, pasta, or cereals. Alkay et al. suggested that the fermentation process involved in making bread, especially sourdough, might influence the bioavailability of certain minerals such as magnesium, potassium, and calcium [37]. Bread’s fermentation process can decrease the levels of phytates, compounds that inhibit mineral absorption, which could result in different mineral absorption dynamics compared with non-fermented grain products like pasta and rice. Moreover, bread, especially when consumed in large quantities, may impact citrate excretion differently from other grains [38]. Citrate is a natural inhibitor of stone formation, and although whole grains might support higher citrate levels due to their magnesium and fiber content, bread and other processed grains might have a less favorable effect on urinary citrate excretion. Finally, in terms of combined nutrient intake, bread is often consumed with other foods like butter, spreads, and meats; these foods can increase caloric density and potentially exacerbate conditions like obesity or metabolic syndrome, which are risk factors for urolithiasis. Grains like rice or oats, which are often eaten as part of a more balanced meal (e.g., with vegetables or lean proteins), may not have the same impact. Therefore, the effect of bread may be multifaceted.

Legumes, soy products, nuts, spinach, and strawberries are rich in oxalate products. In our study, we observed higher prevalence of both urolithiasis and renal colic symptoms in respondents consuming these products. Therefore, our study supports previous findings that high intake of oxalate rich products may induce higher oxalate urine concentration and stone formation of calcium oxalate stones [39]. Nevertheless, an interesting observation emerged from our multivariate analysis. Spinach consumption (specifically ‘once a week’) and strawberry consumption (specifically ‘every day’) were associated with a protective effect against urolithiasis. These discrepancies may be attributed to other factors that may have a protective impact. The oxalate content of spinach can vary significantly based on variety, growth conditions, and season. For instance, Kawazu et al. analyzed 213 spinach varieties and found oxalate levels ranging from 0.76% to 1.8% of fresh weight [40]. This variability suggests that not all spinach consumption contributes equally to oxalate intake. The impact of dietary oxalate on kidney stone formation also depends on oxalate bioavailability and absorption. Although spinach is high in oxalates, the actual absorption can be influenced by other dietary components. For example, consuming calcium-rich foods alongside oxalate-rich foods can reduce oxalate absorption by forming insoluble complexes in the gut [41]. Strawberries, despite containing oxalates, are rich in antioxidants, fiber, and vitamin C. These compounds may contribute to overall kidney health and potentially offset the risks associated with oxalate content. Finally, moderation has a crucial influence. Consuming spinach once a week and strawberries daily, as noted in our study, may provide health benefits without significantly increasing the risk of stone formation. This idea aligns with findings of Salgado et al. who reported that moderate intake of oxalate-containing foods, especially when balanced with other dietary factors, does not substantially elevate kidney stone risk [42]. The protective effect observed might also reflect the benefits of a balanced diet rather than the isolated impact of specific foods [43].

The European Association of Urology Guidelines on Urolithiasis recommend maintaining a generous intake of fluids for the prevention of stone recurrence, with a strong preference for water as the primary fluid source [21]. Indeed, a high intake of fluids containing added sugars is linked to an increased prevalence of urolithiasis [44]. Carbohydrates can alter urine composition by elevating uric acid, calcium, and oxalate, key contributors to kidney stone formation [45]. Fructose is proposed as a key factor mediating the increased risk of kidney stone formation associated with soda consumption [31]. Notably, fruit juices and sugar-sweetened beverages are particularly high in sugars. In our study, respondents who consumed fruit juices or sugar-sweetened beverages had a higher prevalence of both renal colic symptoms and urolithiasis compared with persons who did not consume those items. Importantly, fruit juice consumption emerged as an independent risk factor for renal colic symptoms, whereas sugar-sweetened beverage intake was an independent risk factor for both renal colic symptoms and urolithiasis. The analysis of data from 194,095 participants in the Health Professionals Follow-Up Study, and Nurses’ Health Study I and II by Ferraro et al., with a median follow-up of more than eight years, also indicated significant positive associations with the risk of stone formation for sugar-sweetened cola and sugar-sweetened non-cola, confirming our results [46]. Regarding tea and coffee, the literature presents conflicting findings. Ferraro et al. reported an inverse association between tea and coffee intake and the risk of urolithiasis [46]. This protective effect is suggested to stem from the diuretic properties of these beverages, which may at least partially counteract their hypercalciuric effects [47]. Our findings support the protective effect of tea; individuals who reported tea consumption had a lower prevalence of both urolithiasis and renal colic-like pain symptoms. Moreover, tea consumption was an independent protective factor for urolithiasis. In contrast, in our study coffee consumption was associated with a higher prevalence of both conditions, and coffee was an independent risk factor for urolithiasis. Several factors may help explain this negative association. First, it is important to consider that the method of coffee preparation and consumption can influence its health effects. For instance, unfiltered coffee contains compounds like cafestol and kahweol, which may raise cholesterol levels, a risk factor for stone formation [48]. Additionally, adding sugar or high-fat dairy products could contribute to metabolic changes that increase stone risk. Further, coffee has diuretic properties, potentially leading to increased urine output. If coffee consumption is not balanced with adequate water intake, it might result in dehydration, concentrating urine and promoting stone formation. Finally, genetic predispositions, underlying health conditions, and variations in diet can modify how coffee affects an individual’s stone risk. For example, certain populations might metabolize coffee differently, influencing its impact on kidney stone formation [49].

Fast food and processed products are often high in added sugars and calories and are commonly associated with poor dietary habits. Moreover, these foods typically contain excessive amounts of sodium that can increase urinary calcium excretion and contribute to kidney stone formation. Indeed, our initial findings confirmed that dietary sodium, particularly from added salt, plays a significant role in stone formation [14]. The National Institute of Diabetes and Digestive and Kidney Diseases recommends minimizing the consumption of processed and fast foods as a preventive measure against urolithiasis [50]. Consistent with these concerns, our findings revealed a strong correlation between fast food consumption and both renal colic symptoms and urolithiasis. Notably, daily fast food intake nearly triples the risk of developing urolithiasis. For these reasons, limiting fast food consumption should be considered a key dietary strategy for kidney stone prevention.

A main limitation of our study is its reliance on self-reported data without clinical verification, which may affect accuracy, as noted by Coyne et al. [51]. This introduces a potential recall bias, as participants may not accurately remember or report past symptoms or diagnoses. Some respondents may have misunderstood questions (for example, confusing renal colic–like pain with other conditions), and the survey length limited the inclusion of all relevant factors. Importantly, dietary recommendations can vary depending on stone type, but obviously we could not identify specific stone compositions. Lastly, we do not know whether our findings can be generalized beyond the Central and Eastern European population that we studied, as cultural, socioeconomic, and healthcare system differences may influence the prevalence and perception of these conditions.

## Conclusions

This study presents the first comprehensive, population-based assessment of urolithiasis, renal colic-like pain symptoms, and dietary patterns, including body weight, in Central and Eastern Europe. Our findings indicate that both conditions are influenced by body weight and various dietary factors. These insights may inform health improvement programs and educational campaigns in Poland aimed at reducing the burden of urolithiasis and renal colic-like pain symptoms.

## Supporting information

S1 Table Association of urolithiasis with dietary habits. (DOCX)

S2 Table Association of renal colic-like pain symptoms with dietary habits. (DOCX)

## References

[1] Sorokin I, Mamoulakis C, Miyazawa K, Rodgers A, Talati J, Lotan Y. Epidemiology of stone disease across the world. World J Urol. 2017;35(9):1301–20. doi: 10.1007/s00345-017-2008-6 28213860

[2] Yasui T, Iguchi M, Suzuki S, Kohri K. Prevalence and epidemiological characteristics of urolithiasis in Japan: national trends between 1965 and 2005. Urology. 2008;71(2):209–13. doi: 10.1016/j.urology.2007.09.034 18308085

[3] Indridason OS, Birgisson S, Edvardsson VO, Sigvaldason H, Sigfusson N, Palsson R. Epidemiology of kidney stones in Iceland: a population-based study. Scand J Urol Nephrol. 2006;40(3):215–20. doi: 10.1080/00365590600589898 16809263

[4] Edvardsson VO, Indridason OS, Haraldsson G, Kjartansson O, Palsson R. Temporal trends in the incidence of kidney stone disease. Kidney Int. 2013;83(1):146–52. doi: 10.1038/ki.2012.320 22992468

[5] Zhe M, Hang Z. Nephrolithiasis as a risk factor of chronic kidney disease: a meta-analysis of cohort studies with 4,770,691 participants. Urolithiasis. 2017;45(5):441–8. doi: 10.1007/s00240-016-0938-x 27837248

[6] Shoag J, Halpern J, Goldfarb DS, Eisner BH. Risk of chronic and end stage kidney disease in patients with nephrolithiasis. J Urol. 2014;192(5):1440–5. doi: 10.1016/j.juro.2014.05.117 24929140

[7] Keddis MT, Rule AD. Nephrolithiasis and loss of kidney function. Curr Opin Nephrol Hypertens. 2013;22(4):390–6. doi: 10.1097/MNH.0b013e32836214b9 23736840 PMC4074537

[8] Ní Néill E, Richards HL, Hennessey D, Ryan EM, Fortune DG. Psychological Distress in Patients With Urolithiasis: A Systematic Review and Meta-analysis. J Urol. 2023;209(1):58–70. doi: 10.1097/JU.0000000000003032 36251416

[9] Awedew AF, Han H, Berice BN, Dodge M, Schneider RD, Abbasi-Kangevari M. The global, regional, and national burden of urolithiasis in 204 countries and territories, 2000-2021: a systematic analysis for the Global Burden of Disease Study 2021. EClinicalMedicine. 2024;78. 10.1016/j.eclinm.2024.102924 PMC11618031 39640943

[10] Geraghty RM, Cook P, Walker V, Somani BK. Evaluation of the economic burden of kidney stone disease in the UK: a retrospective cohort study with a mean follow-up of 19 years. BJU Int 2020. 125, 586–94. 31916369 10.1111/bju.14991

[11] Antonelli JA, Maalouf NM, Pearle MS, Lotan Y. Use of the National Health and Nutrition Examination Survey to calculate the impact of obesity and diabetes on cost and prevalence of urolithiasis in 2030. Eur Urol. 2014;66(4):724–9. doi: 10.1016/j.eururo.2014.06.036 25015037 PMC4227394

[12] Bouwman II, Voskamp MJH, Kollen BJ, Nijman RJM, van der Heide WK, Blanker MH. Do lower urinary tract symptoms predict cardiovascular diseases in older men? A systematic review and meta-analysis. World J Urol. 2015;33(12):1911–20. doi: 10.1007/s00345-015-1560-1 25971203 PMC4655016

[13] Choi JWJ, Ford ES, Gao X, Choi HK. Sugar-sweetened soft drinks, diet soft drinks, and serum uric acid level: the Third National Health and Nutrition Examination Survey. Arthritis Rheum. 2008;59(1):109–16. doi: 10.1002/art.23245 18163396

[14] Szymanski J, Chlosta M, Dudek P, Rajwa P, Krajewski W, Bryniarski P, et al. Prevalence, correlates, and treatment behaviors for urolithiasis and renal colic-like pain symptoms at the population level in Poland. Sci Rep. 2025;15(1):10827. doi: 10.1038/s41598-025-95504-x 40155525 PMC11953343

[15] Szymanski J, Rajwa P, Krajewski W, Bryniarski P, Dudek P, Chlosta P. Treatment patterns for urolithiasis and renal colic-like pain symptoms in Poland: The POLSTONE Study. Cent European J Urol. 2025. doi: 10.5173/ceju.2025.0112 PMC12663812 41322464

[16] Glowny Urzad Statystyczny G. Spoleczenstwo informacyjne w Polsce w 2022 r. 2022. https://stat.gov.pl/download/gfx/portalinformacyjny/pl/defaultaktualnosci/5497/2/12/1/spoleczenstwo_informacyjne_w_polsce_w_2022_r.pdf

[17] Chen Y-K, Lin H-C, Chen C-S, Yeh S-D. Seasonal variations in urinary calculi attacks and their association with climate: a population based study. J Urol. 2008;179(2):564–9. doi: 10.1016/j.juro.2007.09.067 18082222

[18] Geraghty RM, Proietti S, Traxer O, Archer M, Somani BK. Worldwide Impact of Warmer Seasons on the Incidence of Renal Colic and Kidney Stone Disease: Evidence from a Systematic Review of Literature. J Endourol. 2017;31(8):729–35. doi: 10.1089/end.2017.0123 28338351

[19] Glowny Urzad Statystyczny (GUS). Narodowy spis powszechny 2021, wyniki ostateczne. 2023. https://stat.gov.pl/spisy-powszechne/nsp-2021/nsp-2021-wyniki-ostateczne/

[20] Glowny Urzad Statystyczny (GUS). Classification of territorial units. Administrative division of Poland types of gminas and urban and rural areas. https://stat.gov.pl/en/regional-statistics/classification-of-territorial-units/administrative-division-of-poland/types-of-gminas-and-urban-and-rural-areas/. 2022. Accessed 2024 December.

[21] European Association of Urology (EAU). Non-oncology guidelines. https://uroweb.org/guideline/urolithiasis/. 2024. Accessed 2024 December.

[22] Fink HA, Wilt TJ, Eidman KE, Garimella PS, MacDonald R, Rutks IR, et al. Medical management to prevent recurrent nephrolithiasis in adults: a systematic review for an American College of Physicians Clinical Guideline. Ann Intern Med. 2013;158(7):535–43. doi: 10.7326/0003-4819-158-7-201304020-00005 23546565

[23] Kocvara R, Plasgura P, Petrík A, Louzenský G, Bartonícková K, Dvorácek J. A prospective study of nonmedical prophylaxis after a first kidney stone. BJU Int. 1999;84(4):393–8. doi: 10.1046/j.1464-410x.1999.00216.x 10468751

[24] Hess B, Mauron H, Ackermann D, Jaeger P. Effects of a “common sense diet” on urinary composition and supersaturation in patients with idiopathic calcium urolithiasis. Eur Urol. 1999;36(2):136–43. doi: 10.1159/000067985 10420035

[25] Siener R, Glatz S, Nicolay C, Hesse A. The role of overweight and obesity in calcium oxalate stone formation. Obes Res. 2004;12(1):106–13. doi: 10.1038/oby.2004.14 14742848

[26] Emami E, Heidari-Soureshjani S, Oroojeni Mohammadjavad A, Sherwin CM. Obesity and the Risk of Developing Kidney Stones: A Systematic Review and Meta-analysis. Iran J Kidney Dis. 2023;17(2):63–72. 37060339

[27] Chen W, Man S, Hong Y, Kadeerhan G, Chen L, Xu Q, et al. Association between metabolically healthy obesity and kidney stones: results from the 2011-2018 National Health and Nutrition Examination Survey. Front Public Health. 2023;11:1103393. doi: 10.3389/fpubh.2023.1103393 37304121 PMC10249726

[28] Cuntz U, Quadflieg N, Voderholzer U. Health Risk and Underweight. Nutrients. 2023;15(14):3262. doi: 10.3390/nu15143262 37513680 PMC10383423

[29] Persaud-Sharma D, Saha S, Trippensee AW. Refeeding Syndrome. StatPearls. 2025. 33232094

[30] Denburg MR, Leonard MB, Jemielita TO, Golden NH, Tasian G, Copelovitch L. Risk of urolithiasis in anorexia nervosa: a population-based cohort study using the health improvement network. Eur Eat Disord Rev. 2017;25(5):406–10. doi: 10.1002/erv.2526 28660717

[31] Ferraro PM, Bargagli M, Trinchieri A, Gambaro G. Risk of Kidney Stones: Influence of Dietary Factors, Dietary Patterns, and Vegetarian-Vegan Diets. Nutrients. 2020;12(3):779. doi: 10.3390/nu12030779 32183500 PMC7146511

[32] Tracy CR, Best S, Bagrodia A, Poindexter JR, Adams-Huet B, Sakhaee K, et al. Animal protein and the risk of kidney stones: a comparative metabolic study of animal protein sources. J Urol. 2014;192(1):137–41. doi: 10.1016/j.juro.2014.01.093 24518789

[33] Reddy ST, Wang C-Y, Sakhaee K, Brinkley L, Pak CYC. Effect of low-carbohydrate high-protein diets on acid-base balance, stone-forming propensity, and calcium metabolism. Am J Kidney Dis. 2002;40(2):265–74. doi: 10.1053/ajkd.2002.34504 12148098

[34] Martini S, Conte A, Tagliazucchi D. Comparative peptidomic profile and bioactivities of cooked beef, pork, chicken and turkey meat after in vitro gastro-intestinal digestion. J Proteomics. 2019;208:103500. doi: 10.1016/j.jprot.2019.103500 31454557

[35] Best S, Tracy C, Bagrodia A, Poindexter J, Adams-Huet B, Sakhaee K, et al. 2147 effect of various animal protein sources on urinary stone risk factors. J Urology. 2011;185(4S). doi: 10.1016/j.juro.2011.02.2358

[36] Shabani E, Khorshidi A, Sayehmiri K, Moradi K, Nabi Abdolyousefi E. The effect of nutritional factors on urolithiasis: A case-control study. J Med Life. 2023;16(7):1062–9. doi: 10.25122/jml-2022-0321 37900086 PMC10600667

[37] Alkay Z, Falah F, Cankurt H, Dertli E. Exploring the Nutritional Impact of Sourdough Fermentation: Its Mechanisms and Functional Potential. Foods. 2024;13(11):1732. doi: 10.3390/foods13111732 38890959 PMC11172170

[38] D’Alessandro C, Ferraro PM, Cianchi C, Barsotti M, Gambaro G, Cupisti A. Which diet for calcium stone patients: a real-world approach to preventive care. Nutrients. 2019;11(5):1182. doi: 10.3390/nu11051182 31137803 PMC6566930

[39] Mitchell T, Kumar P, Reddy T, Wood KD, Knight J, Assimos DG, et al. Dietary oxalate and kidney stone formation. Am J Physiol Renal Physiol. 2019;316(3):F409–13. doi: 10.1152/ajprenal.00373.2018 30566003 PMC6459305

[40] Kawazu Y, Okimura M, Ishii T, Yui S. Varietal and seasonal differences in oxalate content of spinach. Scientia Horticulturae. 2003;97(3–4):203–10. doi: 10.1016/s0304-4238(02)00154-1

[41] Massey LK, Roman-Smith H, Sutton RA. Effect of dietary oxalate and calcium on urinary oxalate and risk of formation of calcium oxalate kidney stones. J Am Diet Assoc. 1993;93(8):901–6. doi: 10.1016/0002-8223(93)91530-4 8335871

[42] Salgado N, Silva MA, Figueira ME, Costa HS, Albuquerque TG. Oxalate in Foods: Extraction Conditions, Analytical Methods, Occurrence, and Health Implications. Foods. 2023;12. 10.3390/foods12173201 PMC10486698 37685134

[43] Sorensen MD, Hsi RS, Chi T, Shara N, Wactawski-Wende J, Kahn AJ, et al. Dietary intake of fiber, fruit and vegetables decreases the risk of incident kidney stones in women: a Women’s Health Initiative report. J Urol. 2014;192(6):1694–9. doi: 10.1016/j.juro.2014.05.086 24859445 PMC4241174

[44] Yin S, Yang Z, Zhu P, Du Z, Yu X, Tang T, et al. Association between added sugars and kidney stones in U.S. adults: data from National Health and Nutrition Examination Survey 2007-2018. Front Nutr. 2023;10:1226082. doi: 10.3389/fnut.2023.1226082 37599678 PMC10436224

[45] Siener R. Nutrition and kidney stone disease. Nutrients. 2021;13. 10.3390/nu13061917 PMC8229448 34204863

[46] Ferraro PM, Taylor EN, Gambaro G, Curhan GC. Soda and other beverages and the risk of kidney stones. Clin J Am Soc Nephrol. 2013;8(8):1389–95. doi: 10.2215/CJN.11661112 23676355 PMC3731916

[47] Barghouthy Y, Corrales M, Somani B. The Relationship between Modern Fad Diets and Kidney Stone Disease: A Systematic Review of Literature. Nutrients. 2021;13(12):4270. doi: 10.3390/nu13124270 34959822 PMC8708871

[48] Silva JA, Borges N, Santos A, Alves A. Method Validation for Cafestol and Kahweol Quantification in Coffee Brews by HPLC-DAD. Food Anal Methods. 2012;5(6):1404–10. doi: 10.1007/s12161-012-9387-5

[49] Geng J, Qiu Y, Kang Z, Li Y, Li J, Liao R, et al. The association between caffeine intake and risk of kidney stones: A population-based study. Front Nutr. 2022;9:935820. doi: 10.3389/fnut.2022.935820 36299992 PMC9589282

[50] National Institute of Diabetes and Digestive and Kidney Diseases (NIDDK). Eating, diet, & nutrition for kidney stones - NIDDK. https://www.niddk.nih.gov/health-information/urologic-diseases/kidney-stones/eating-diet-nutrition. 2024.

[51] Coyne KS, Kaplan SA, Chapple CR, Sexton CC, Kopp ZS, Bush EN, et al. Risk factors and comorbid conditions associated with lower urinary tract symptoms: EpiLUTS. BJU Int. 2009;103 Suppl 3:24–32. doi: 10.1111/j.1464-410X.2009.08438.x 19302499

