# Supplementary material for: Diet and stones: Associations from a large, population-representative study of urolithiasis and renal colic-like pain symptoms in Poland
Source: PLoS One. 2026 Feb 3;21(2):e0333733. doi: 10.1371/journal.pone.0333733 (PMC12867237; doi:10.1371/journal.pone.0333733)
Supplement: S1 Table — (DOCX) [file pone.0333733.s001.docx]

S1 Table. Association of urolithiasis with dietary habits

| **Parameter** | **Group** | **Urolithiasis** | | **p** |
| --- | --- | --- | --- | --- |
|  |  | **No** | **Yes** |  |
| Beef | Never (N=1422) | 1266 (89.03%) | 156 (10.97%) | p<0.001 * |
|  | Rarely (N=5588) | 4900 (87.69%) | 688 (12.31%) |  |
|  | Once in a week (N=2194) | 1927 (87.83%) | 267 (12.17%) |  |
|  | Every other day (N=489) | 406 (83.03%) | 83 (16.97%) |  |
|  | Every day(N=336) | 241 (71.73%) | 95 (28.27%) |  |
| Pork | Never (N=405) | 343 (84.69%) | 62 (15.31%) | p<0.001 * |
|  | Rarely (N=2126) | 1880 (88.43%) | 246 (11.57%) |  |
|  | Once in a week (N=4655) | 4101 (88.10%) | 554 (11.90%) |  |
|  | Every other day (N=2427) | 2069 (85.25%) | 358 (14.75%) |  |
|  | Every day (N=416) | 347 (83.41%) | 69 (16.59%) |  |
| Poultry | Never (N=249) | 201 (80.72%) | 48 (19.28%) | p<0.001 * |
|  | Rarely (N=885) | 762 (86.10%) | 123 (13.90%) |  |
|  | Once in a week (N=4457) | 3930 (88.18%) | 527 (11.82%) |  |
|  | Every other day (N=3876) | 3380 (87.20%) | 496 (12.80%) |  |
|  | Every day (N=562) | 467 (83.10%) | 95 (16.90%) |  |
| Processed meats (cold cuts, sausages, frankfurters, pâtés, canned meats) | Never (N=333) | 278 (83.48%) | 55 (16.52%) | p=0.005 * |
|  | Rarely (N=1184) | 1006 (84.97%) | 178 (15.03%) |  |
|  | Once in a week (N=2258) | 1955 (86.58%) | 303 (13.42%) |  |
|  | Every other day (N=3489) | 3052 (87.47%) | 437 (12.53%) |  |
|  | Every day (N=2765) | 2449 (88.57%) | 316 (11.43%) |  |
| Dairy | Never (N=97) | 75 (77.32%) | 22 (22.68%) | p=0.011 * |
|  | Rarely (N=418) | 357 (85.41%) | 61 (14.59%) |  |
|  | Once in a week (N=1549) | 1333 (86.06%) | 216 (13.94%) |  |
|  | Every other day (N=3237) | 2823 (87.21%) | 414 (12.79%) |  |
|  | Every day (N=4728) | 4152 (87.82%) | 576 (12.18%) |  |
| Grain products | Never (N=238) | 201 (84.45%) | 37 (15.55%) | p=0.615 |
|  | Rarely (N=1433) | 1240 (86.53%) | 193 (13.47%) |  |
|  | Once in a week (N=2515) | 2204 (87.63%) | 311 (12.37%) |  |
|  | Every other day (N=2469) | 2149 (87.04%) | 320 (12.96%) |  |
|  | Every day (N=3374) | 2946 (87.31%) | 428 (12.69%) |  |
| White bread | Never (N=560) | 481 (85.89%) | 79 (14.11%) | p<0.001 * |
|  | Rarely (N=1419) | 1225 (86.33%) | 194 (13.67%) |  |
|  | Once in a week (N=1270) | 1066 (83.94%) | 204 (16.06%) |  |
|  | Every other day (N=2116) | 1819 (85.96%) | 297 (14.04%) |  |
|  | Every day (N=4664) | 4149 (88.96%) | 515 (11.04%) |  |
| Dark bread | Never (N=685) | 603 (88.03%) | 82 (11.97%) | p<0.001 * |
|  | Rarely (N=2733) | 2449 (89.61%) | 284 (10.39%) |  |
|  | Once in a week (N=2025) | 1776 (87.70%) | 249 (12.30%) |  |
|  | Every other day (N=2303) | 1979 (85.93%) | 324 (14.07%) |  |
|  | Every day (N=2283) | 1933 (84.67%) | 350 (15.33%) |  |
| Legumes | Never (N=603) | 546 (90.55%) | 57 (9.45%) | p<0.001 * |
|  | Rarely (N=4750) | 4197 (88.36%) | 553 (11.64%) |  |
|  | Once in a week (N=3625) | 3166 (87.34%) | 459 (12.66%) |  |
|  | Every other day (N=818) | 665 (81.30%) | 153 (18.70%) |  |
|  | Every day (N=233) | 166 (71.24%) | 67 (28.76%) |  |
| Soy products | Never (N=3703) | 3282 (88.63%) | 421 (11.37%) | p<0.001 * |
|  | Rarely (N=4480) | 3971 (88.64%) | 509 (11.36%) |  |
|  | Once in a week (N=1236) | 1022 (82.69%) | 214 (17.31%) |  |
|  | Every other day (N=435) | 348 (80.00%) | 87 (20.00%) |  |
|  | Every day (N=175) | 117 (66.86%) | 58 (33.14%) |  |
| Fresh Fruits | Never (N=108) | 90 (83.33%) | 18 (16.67%) | p=0.161 |
|  | Rarely (N=902) | 796 (88.25%) | 106 (11.75%) |  |
|  | Once in a week (N=1998) | 1745 (87.34%) | 253 (12.66%) |  |
|  | Every other day (N=2868) | 2523 (87.97%) | 345 (12.03%) |  |
|  | Every day (N=4153) | 3586 (86.35%) | 567 (13.65%) |  |
| Fresh vegetables | Never (N=95) | 79 (83.16%) | 16 (16.84%) | p=0.293 |
|  | Rarely (N=849) | 742 (87.40%) | 107 (12.60%) |  |
|  | Once in a week (N=2097) | 1823 (86.93%) | 274 (13.07%) |  |
|  | Every other day (N=3281) | 2888 (88.02%) | 393 (11.98%) |  |
|  | Every day (N=3707) | 3208 (86.54%) | 499 (13.46%) |  |
| Nuts | Never (N=714) | 621 (86.97%) | 93 (13.03%) | p<0.001 * |
|  | Rarely (N=4058) | 3584 (88.32%) | 474 (11.68%) |  |
|  | Once in a week (N=3048) | 2658 (87.20%) | 390 (12.80%) |  |
|  | Every other day (N=1321) | 1142 (86.45%) | 179 (13.55%) |  |
|  | Every day (N=888) | 735 (82.77%) | 153 (17.23%) |  |
| Cocoa. Chocolate | Never (N=452) | 384 (84.96%) | 68 (15.04%) | p=0.284 |
|  | Rarely (N=3335) | 2930 (87.86%) | 405 (12.14%) |  |
|  | Once in a week (N=3657) | 3182 (87.01%) | 475 (12.99%) |  |
|  | Every other day (N=1788) | 1561 (87.30%) | 227 (12.70%) |  |
|  | Every day (N=797) | 683 (85.70%) | 114 (14.30%) |  |
| Highly-processed sweets | Never (N=567) | 483 (85.19%) | 84 (14.81%) | p=0.23 |
|  | Rarely (N=3056) | 2693 (88.12%) | 363 (11.88%) |  |
|  | Once in a week (N=3461) | 3003 (86.77%) | 458 (13.23%) |  |
|  | Every other day (N=2094) | 1827 (87.25%) | 267 (12.75%) |  |
|  | Every day (N=851) | 734 (86.25%) | 117 (13.75%) |  |
| Spinach | Never (N=2282) | 1989 (87.16%) | 293 (12.84%) | p<0.001 * |
|  | Rarely (N=5038) | 4448 (88.29%) | 590 (11.71%) |  |
|  | Once in a week (N=2099) | 1823 (86.85%) | 276 (13.15%) |  |
|  | Every other day (N=466) | 379 (81.33%) | 87 (18.67%) |  |
|  | Every day (N=144) | 101 (70.14%) | 43 (29.86%) |  |
| Strawberries | Never (N=463) | 397 (85.75%) | 66 (14.25%) | p<0.001 * |
|  | Rarely (N=7002) | 6192 (88.43%) | 810 (11.57%) |  |
|  | Once in a week (N=1744) | 1484 (85.09%) | 260 (14.91%) |  |
|  | Every other day (N=564) | 464 (82.27%) | 100 (17.73%) |  |
|  | Every day (N=256) | 203 (79.30%) | 53 (20.70%) |  |
| Fruit juices | Never (N=686) | 596 (86.88%) | 90 (13.12%) | p=0.004 * |
|  | Rarely (N=3309) | 2934 (88.67%) | 375 (11.33%) |  |
|  | Once in a week (N=3043) | 2653 (87.18%) | 390 (12.82%) |  |
|  | Every other day (N=1939) | 1667 (85.97%) | 272 (14.03%) |  |
|  | Every day (N=1052) | 890 (84.60%) | 162 (15.40%) |  |
| Sweet beverages | Never (N=2339) | 2032 (86.87%) | 307 (13.13%) | p=0.008 * |
|  | Rarely (N=3737) | 3310 (88.57%) | 427 (11.43%) |  |
|  | Once in a week (N=2088) | 1809 (86.64%) | 279 (13.36%) |  |
|  | Every other day (N=1084) | 923 (85.15%) | 161 (14.85%) |  |
|  | Every day (N=781) | 666 (85.28%) | 115 (14.72%) |  |
| Coffee | Never (N=972) | 877 (90.23%) | 95 (9.77%) | p<0.001 * |
|  | Rarely (N=661) | 584 (88.35%) | 77 (11.65%) |  |
|  | Once in a week (N=635) | 506 (79.69%) | 129 (20.31%) |  |
|  | Every other day (N=787) | 657 (83.48%) | 130 (16.52%) |  |
|  | Every day (N=6974) | 6116 (87.70%) | 858 (12.30%) |  |
| Tea | Never (N=314) | 258 (82.17%) | 56 (17.83%) | p=0.002 * |
|  | Rarely (N=876) | 768 (87.67%) | 108 (12.33%) |  |
|  | Once in a week (N=902) | 761 (84.37%) | 141 (15.63%) |  |
|  | Every other day (N=1268) | 1093 (86.20%) | 175 (13.80%) |  |
|  | Every day (N=6669) | 5860 (87.87%) | 809 (12.13%) |  |
| Instant meals | Never (N=3730) | 3207 (85.98%) | 523 (14.02%) | p<0.001 * |
|  | Rarely (N=4080) | 3639 (89.19%) | 441 (10.81%) |  |
|  | Once in a week (N=1544) | 1354 (87.69%) | 190 (12.31%) |  |
|  | Every other day (N=478) | 397 (83.05%) | 81 (16.95%) |  |
|  | Every day (N=197) | 143 (72.59%) | 54 (27.41%) |  |
| Fast food | Never (N=2301) | 1971 (85.66%) | 330 (14.34%) | p<0.001 * |
|  | Rarely (N=5409) | 4805 (88.83%) | 604 (11.17%) |  |
|  | Once in a week (N=1763) | 1539 (87.29%) | 224 (12.71%) |  |
|  | Every other day (N=429) | 346 (80.65%) | 83 (19.35%) |  |
|  | Every day (N=127) | 79 (62.20%) | 48 (37.80%) |  |

p - chi-square test or Fisher’s exact test

* statistically significant difference (p<0.05)
