# Supplementary material for: Diet and stones: Associations from a large, population-representative study of urolithiasis and renal colic-like pain symptoms in Poland
Source: PLoS One. 2026 Feb 3;21(2):e0333733. doi: 10.1371/journal.pone.0333733 (PMC12867237; doi:10.1371/journal.pone.0333733)
Supplement: S2 Table — (DOCX) [file pone.0333733.s002.docx]

S2 Table. Association of renal colic-like pain symptoms with dietary habits

| **Parameter** | **Group** | **Renal colic-like pain symptoms** | | **p** |
| --- | --- | --- | --- | --- |
|  |  | **No** | **Yes** |  |
| Beef | Never (N=1422) | 857 (60.27%) | 565 (39.73%) | p<0.001 * |
|  | Rarely (N=5588) | 3295 (58.97%) | 2293 (41.03%) |  |
|  | Once in a week (N=2194) | 1205 (54.92%) | 989 (45.08%) |  |
|  | Every other day (N=489) | 217 (44.38%) | 272 (55.62%) |  |
|  | Every day (N=336) | 138 (41.07%) | 198 (58.93%) |  |
| Pork | Never (N=405) | 252 (62.22%) | 153 (37.78%) | p<0.001 * |
|  | Rarely (N=2126) | 1283 (60.35%) | 843 (39.65%) |  |
|  | Once in a week (N=4655) | 2665 (57.25%) | 1990 (42.75%) |  |
|  | Every other day(N=2427) | 1302 (53.65%) | 1125 (46.35%) |  |
|  | Every day (N=416) | 210 (50.48%) | 206 (49.52%) |  |
| Poultry | Never(N=249) | 158 (63.45%) | 91 (36.55%) | p<0.001 * |
|  | Rarely (N=885) | 520 (58.76%) | 365 (41.24%) |  |
|  | Once in a week (N=4457) | 2632 (59.05%) | 1825 (40.95%) |  |
|  | Every other day (N=3876) | 2152 (55.52%) | 1724 (44.48%) |  |
|  | Every day (N=562) | 250 (44.48%) | 312 (55.52%) |  |
| Processed meats (cold cuts, sausages, frankfurters, pâtés, canned meats) | Never (N=333) | 222 (66.67%) | 111 (33.33%) | p<0.001 * |
|  | Rarely (N=1184) | 700 (59.12%) | 484 (40.88%) |  |
|  | Once in a week (N=2258) | 1293 (57.26%) | 965 (42.74%) |  |
|  | Every other day (N=3489) | 1994 (57.15%) | 1495 (42.85%) |  |
|  | Every day (N=2765) | 1503 (54.36%) | 1262 (45.64%) |  |
| Dairy | Never (N=97) | 52 (53.61%) | 45 (46.39%) | p=0.183 |
|  | Rarely (N=418) | 231 (55.26%) | 187 (44.74%) |  |
|  | Once in a week (N=1549) | 846 (54.62%) | 703 (45.38%) |  |
|  | Every other day (N=3237) | 1881 (58.11%) | 1356 (41.89%) |  |
|  | Every day (N=4728) | 2702 (57.15%) | 2026 (42.85%) |  |
| Grain products | Never (N=238) | 133 (55.88%) | 105 (44.12%) | p=0.036 * |
|  | Rarely (N=1433) | 814 (56.80%) | 619 (43.20%) |  |
|  | Once in a week (N=2515) | 1388 (55.19%) | 1127 (44.81%) |  |
|  | Every other day (N=2469) | 1384 (56.06%) | 1085 (43.94%) |  |
|  | Every day (N=3374) | 1993 (59.07%) | 1381 (40.93%) |  |
| White bread | Never (N=560) | 347 (61.96%) | 213 (38.04%) | p<0.001 * |
|  | Rarely (N=1419) | 881 (62.09%) | 538 (37.91%) |  |
|  | Once in a week (N=1270) | 675 (53.15%) | 595 (46.85%) |  |
|  | Every other day (N=2116) | 1213 (57.33%) | 903 (42.67%) |  |
|  | Every day (N=4664) | 2596 (55.66%) | 2068 (44.34%) |  |
| Dark bread | Never (N=685) | 384 (56.06%) | 301 (43.94%) | p<0.001 * |
|  | Rarely (N=2733) | 1632 (59.71%) | 1101 (40.29%) |  |
|  | Once in a week (N=2025) | 1069 (52.79%) | 956 (47.21%) |  |
|  | Every other day (N=2303) | 1301 (56.49%) | 1002 (43.51%) |  |
|  | Every day (N=2283) | 1326 (58.08%) | 957 (41.92%) |  |
| Legumes | Never (N=603) | 343 (56.88%) | 260 (43.12%) | p<0.001 * |
|  | Rarely (N=4750) | 2783 (58.59%) | 1967 (41.41%) |  |
|  | Once in a week (N=3625) | 2095 (57.79%) | 1530 (42.21%) |  |
|  | Every other day (N=818) | 407 (49.76%) | 411 (50.24%) |  |
|  | Every day (N=233) | 84 (36.05%) | 149 (63.95%) |  |
| Soy products | Never (N=3703) | 2222 (60.01%) | 1481 (39.99%) | p<0.001 * |
|  | Rarely (N=4480) | 2607 (58.19%) | 1873 (41.81%) |  |
|  | Once in a week (N=1236) | 620 (50.16%) | 616 (49.84%) |  |
|  | Every other day (N=435) | 203 (46.67%) | 232 (53.33%) |  |
|  | Every day (N=175) | 60 (34.29%) | 115 (65.71%) |  |
| Fresh Fruits | Never (N=108) | 57 (52.78%) | 51 (47.22%) | p=0.022 * |
|  | Rarely (N=902) | 506 (56.10%) | 396 (43.90%) |  |
|  | Once in a week (N=1998) | 1083 (54.20%) | 915 (45.80%) |  |
|  | Every other day (N=2868) | 1635 (57.01%) | 1233 (42.99%) |  |
|  | Every day (N=4153) | 2431 (58.54%) | 1722 (41.46%) |  |
| Fresh vegetables | Never (N=95) | 53 (55.79%) | 42 (44.21%) | p=0.146 |
|  | Rarely (N=849) | 468 (55.12%) | 381 (44.88%) |  |
|  | Once in a week (N=2097) | 1155 (55.08%) | 942 (44.92%) |  |
|  | Every other day (N=3281) | 1877 (57.21%) | 1404 (42.79%) |  |
|  | Every day (N=3707) | 2159 (58.24%) | 1548 (41.76%) |  |
| Nuts | Never (N=714) | 404 (56.58%) | 310 (43.42%) | p=0.816 |
|  | Rarely (N=4058) | 2290 (56.43%) | 1768 (43.57%) |  |
|  | Once in a week (N=3048) | 1736 (56.96%) | 1312 (43.04%) |  |
|  | Every other day (N=1321) | 768 (58.14%) | 553 (41.86%) |  |
|  | Every day (N=888) | 514 (57.88%) | 374 (42.12%) |  |
| Cocoa. Chocolate | Never (N=452) | 255 (56.42%) | 197 (43.58%) | p=0.098 |
|  | Rarely (N=3335) | 1952 (58.53%) | 1383 (41.47%) |  |
|  | Once in a week (N=3657) | 2079 (56.85%) | 1578 (43.15%) |  |
|  | Every other day (N=1788) | 998 (55.82%) | 790 (44.18%) |  |
|  | Every day (N=797) | 428 (53.70%) | 369 (46.30%) |  |
| Highly-processed sweets | Never (N=567) | 339 (59.79%) | 228 (40.21%) | p<0.001 * |
|  | Rarely (N=3056) | 1848 (60.47%) | 1208 (39.53%) |  |
|  | Once in a week(N=3461) | 1967 (56.83%) | 1494 (43.17%) |  |
|  | Every other day (N=2094) | 1139 (54.39%) | 955 (45.61%) |  |
|  | Every day (N=851) | 419 (49.24%) | 432 (50.76%) |  |
| Spinach | Never (N=2282) | 1304 (57.14%) | 978 (42.86%) | p<0.001 * |
|  | Rarely (N=5038) | 2962 (58.79%) | 2076 (41.21%) |  |
|  | Once in a week (N=2099) | 1183 (56.36%) | 916 (43.64%) |  |
|  | Every other day (N=466) | 210 (45.06%) | 256 (54.94%) |  |
|  | Every day (N=144) | 53 (36.81%) | 91 (63.19%) |  |
| Strawberries | Never (N=463) | 259 (55.94%) | 204 (44.06%) | p<0.001 * |
|  | Rarely (N=7002) | 4075 (58.20%) | 2927 (41.80%) |  |
|  | Once in a week (N=1744) | 975 (55.91%) | 769 (44.09%) |  |
|  | Every other day (N=564) | 279 (49.47%) | 285 (50.53%) |  |
|  | Every day (N=256) | 124 (48.44%) | 132 (51.56%) |  |
| Fruit juices | Never (N=686) | 439 (63.99%) | 247 (36.01%) | p<0.001 * |
|  | Rarely (N=3309) | 1974 (59.66%) | 1335 (40.34%) |  |
|  | Once in a week (N=3043) | 1726 (56.72%) | 1317 (43.28%) |  |
|  | Every other day (N=1939) | 1026 (52.91%) | 913 (47.09%) |  |
|  | Every day (N=1052) | 547 (52.00%) | 505 (48.00%) |  |
| Sweet beverages | Never (N=2339) | 1492 (63.79%) | 847 (36.21%) | p<0.001 * |
|  | Rarely (N=3737) | 2213 (59.22%) | 1524 (40.78%) |  |
|  | Once in a week (N=2088) | 1125 (53.88%) | 963 (46.12%) |  |
|  | Every other day (N=1084) | 531 (48.99%) | 553 (51.01%) |  |
|  | Every day (N=781) | 351 (44.94%) | 430 (55.06%) |  |
| Coffee | Never (N=972) | 595 (61.21%) | 377 (38.79%) | p<0.001 * |
|  | Rarely (N=661) | 380 (57.49%) | 281 (42.51%) |  |
|  | Once in a week (N=635) | 308 (48.50%) | 327 (51.50%) |  |
|  | Every other day (N=787) | 404 (51.33%) | 383 (48.67%) |  |
|  | Every day (N=6974) | 4025 (57.71%) | 2949 (42.29%) |  |
| Tea | Never (N=314) | 180 (57.32%) | 134 (42.68%) | p<0.001 * |
|  | Rarely (N=876) | 488 (55.71%) | 388 (44.29%) |  |
|  | Once in a week (N=902) | 472 (52.33%) | 430 (47.67%) |  |
|  | Every other day (N=1268) | 669 (52.76%) | 599 (47.24%) |  |
|  | Every day(N=6669) | 3903 (58.52%) | 2766 (41.48%) |  |
| Instant meals | Never (N=3730) | 2371 (63.57%) | 1359 (36.43%) | p<0.001 * |
|  | Rarely (N=4080) | 2310 (56.62%) | 1770 (43.38%) |  |
|  | Once in a week (N=1544) | 776 (50.26%) | 768 (49.74%) |  |
|  | Every other day (N=478) | 184 (38.49%) | 294 (61.51%) |  |
|  | Every day (N=197) | 71 (36.04%) | 126 (63.96%) |  |
| Fast food | Never (N=2301) | 1498 (65.10%) | 803 (34.90%) | p<0.001 * |
|  | Rarely(N=5409) | 3117 (57.63%) | 2292 (42.37%) |  |
|  | Once in a week (N=1763) | 892 (50.60%) | 871 (49.40%) |  |
|  | Every other day (N=429) | 168 (39.16%) | 261 (60.84%) |  |
|  | Every day (N=127) | 37 (29.13%) | 90 (70.87%) |  |

p - chi-square test or Fisher’s exact test

* statistically significant difference (p<0.05)
